# Understanding the Impact of Cultivar, Seed Origin, and Substrate on Bacterial Diversity of the Sugar Beet Rhizosphere and Suppression of Soil-Borne Pathogens

**DOI:** 10.3389/fpls.2020.560869

**Published:** 2020-09-30

**Authors:** Adrian Wolfgang, Christin Zachow, Henry Müller, Alfred Grand, Nora Temme, Ralf Tilcher, Gabriele Berg

**Affiliations:** ^1^ Austrian Centre of Industrial Biotechnology (ACIB GmbH), Graz, Austria; ^2^ Institute of Environmental Biotechnology, Graz University of Technology, Graz, Austria; ^3^ BioTenzz GmbH, Graz, Austria; ^4^ VERMIGRAND Naturprodukte GmbH, Absdorf, Austria; ^5^ KWS SAAT SE & Co. KGaA, Einbeck, Germany

**Keywords:** sugar beet cultivars, seed microbiome, root microbiome, vermicompost, biocontrol, breeding, *Pseudomonas poae* RE*1-1-14

## Abstract

The rhizosphere microbiome is crucial for plant health, especially for preventing roots from being infected by soil-borne pathogens. Microbiota-mediated pathogen response in the soil-root interface may hold the key for microbiome-based control strategies of phytopathogens. We studied the pathosystem sugar beet—late sugar beet root rot caused by *Rhizoctonia solani* in an integrative design of combining *in vitro* and *in vivo* (greenhouse and field) trials. We used five different cultivars originating from two propagation sites (France, Italy) with different degrees of susceptibility towards *R. solani* (two susceptible, one moderately tolerant and two cultivars with partial resistance). Analyzing bacterial communities in seeds and roots grown under different conditions by 16S rRNA amplicon sequencing, we found site-, cultivar-, and microhabitat-specific amplicon sequences variants (ASV) as well as a seed core microbiome shared between all sugar beet cultivars (121 ASVs representing 80%–91% relative abundance). In general, cultivar-specific differences in the bacterial communities were more pronounced in seeds than in roots. Seeds of *Rhizoctonia*-tolerant cultivars contain a higher relative abundance of the genera *Paenibacillus*, *Kosakonia*, and *Enterobacter*, while *Gaiellales, Rhizobiales*, and *Kosakonia* were enhanced in responsive rhizospheres. These results indicate a correlation between bacterial seed endophytes and *Rhizoctonia*-tolerant cultivars. Root communities are mainly substrate-derived but also comprise taxa exclusively derived from seeds. Interestingly, the signature of *Pseudomonas poae* Re*1-1-14, a well-studied sugar-beet specific biocontrol agent, was frequently found and in higher relative abundances in *Rhizoctonia*-tolerant than in susceptible cultivars. For microbiome management, we introduced microbial inoculants (consortia) and microbiome transplants (vermicompost) in greenhouse and field trials; both can modulate the rhizosphere and mediate tolerance towards late sugar beet root rot. Both, seeds and soil, provide specific beneficial bacteria for rhizosphere assembly and microbiota-mediated pathogen tolerance. This can be translated into microbiome management strategies for plant and ecosystem health.

## Introduction

Developing concepts for microbiome-based crop management strategies is challenging due to the multi-fold interactions in these complex systems. It further requires a deep scientific understanding of microbial community dynamics. Microbiome network structure and microbial diversity in the rhizosphere of plants are linked with tolerance towards pathogen invasion (van Elsas et al., 2012; Berg et al., 2017). The rhizosphere is the critical soil-plant interface for resource exchange and interaction between the plant and soil microbiota (Weller et al., 2002; Philippot et al., 2013). The crucial involvement of rhizosphere-associated microbiota for growth promotion and stress tolerance in crops is known for more than a century (Hiltner, 1904), but was impressively accelerated by the progresses in omics-technologies (Mendes et al., 2012; Raaijmakers and Mazzola, 2016). Due to the importance of microbial rhizosphere assembly for plant health, it was intensively studied in the last decades (Berendsen et al., 2012; Bakker et al., 2013). Diverse factors shaping the microbial rhizosphere community have been identified, with plant genotype and soil traits as the most important determining factors (Berg and Smalla, 2009). However, both plant genotype as well as soil quality, were strongly changed in the period of increasingly intensified agriculture. This resulted in changes in plant-associated microbial communities and reduced indigenous antagonistic potential towards plant pathogens, especially towards soil-borne pathogens (Cardinale et al., 2015; Perez-Jaramillo et al., 2017; Banerjee et al., 2019). Crops often lack clear genetic resistance to soil-borne pathogens. Cook and colleagues postulated already in 1995 that plants could compensate this by recruiting antagonists of pathogens from the soil microbiome. This was now evidenced by omics-technologies (Mendes et al., 2012; Carrión et al., 2019). Although, there are first studies indicating that breeding for resistance towards soil-borne pathogens unintentionally shaped the structure and function of the rhizosphere microbiome, e.g. in the pathosystem common bean (*Phaseolus vulgaris*)—*Fusarium oxysporum* (Mendes et al., 2018a; Mendes et al., 2018b), a generalized relationship between the rhizosphere microbiome structure and plant immunity/tolerance could not be established yet.

Seeds were identified as carrier and ideal target for rhizosphere’s microbiome management (Berg and Raaijmakers, 2018). During the last years, the vertical transmission *via* seed or propagule endophytes has been described for many plants (e.g. Johnston-Monje et al., 2016; Klaedtke et al., 2016; Bergna et al., 2018). However, the role of seed endophytes and their interplay with soil microorganisms in rhizosphere assembly and microbiota-mediated pathogen response is not yet understood (Berg and Raaijmakers, 2018). Furthermore, many basic insights f.e. about the impact of propagation site on seed endophyte assemply are still missing (Bergna et al., 2018). Seed endophytes play an important role in the respective plant-pathogen interactions (Shade et al., 2017). To what extent microbial seed communities are conserved or otherwise shaped by the propagation sites’ soil communities may be important for pathogen susceptibility of the next plant generation. Furthermore, only seed endophytes that survive the dynamic process of germination will be represented in the endophytic community of the seedling and its rhizosphere (Shade et al., 2017).

Sugar beet is an interesting model crop for microbiome studies (Zachow et al., 2008; Mendes et al., 2012; Kusstascher et al., 2019a; Kusstascher et al., 2019b) known for their genome and breeding history (Würschum et al., 2013; Dohm et al., 2014). Sugar beet (*Beta vulgaris* L.) is an important root crop and the main source of sucrose in moderate climates. It is grown on approximately 4.8*10^6^ hectares, resulting in 2.7*10^8^ tonnes yield in 2018, with Russia and France being the main producers worldwide (FAOSTAT, 2019). One of the major pathogens in sugar beet is the fungus *Rhizoctonia solani* J.G.KÜHN [teleomorph: *Thanatephorus cucumeris* (A.B.Frank) Donk], which causes a variety of different plant diseases and has a broad host range (Ogoshi, 1996). Especially the late root rot, a disease caused by *R. solani* of the anastomosis group AG2-2IIIB leads locally to high yield losses over 50%, and is estimated to affect 5%–10% of the acreage in Europe and over 24% in the United States (Büttner et al., 2004; Jacobsen, 2006). Although partially *Rhizoctonia*-resistant or -tolerant sugar beet cultivars are commercially available, they usually are less productive or lack resistance/tolerance towards other diseases (Jacobsen, 2006). Therefore, microbiome-based disease management may be an interesting alternative in the future. Microbiomes can be managed either directly by applying microbiome transplants; single or mixed microbes with bioactive properties; or microbiota-active metabolites, or indirectly by changing environmental conditions in a way that microbiomes also shift their structure and function from dysbiosis into a healthy state (Berg, 2009). One possible approach to directly shape rhizosphere communities is using vermicompost as microbiome transplant. Vermicompost is a biofertilizing substrate produced by earthworms, which are one of the key taxa for soil functionality (Drake and Horn, 2007; Singh et al., 2020). Properties of vermicompost as disease-suppressing microbiome transplant are promising, since it already showed suppression of *Rhizoctonia* in cucumber (Simsek Ersahin et al., 2009).

This study focuses on the origin of root microbiota in general and for *Rhizoctonia* tolerance in particular as well as potential microbiome-based biocontrol options. Microbiome modulation approaches consisted of either bacterial inoculant (three strains belonging to *Pseudomonas* and *Serratia*) or microbiome transplant (vermicompost) application. We investigated the following hypotheses: I) Most seed endophytes of sugar beet survive until the process of germination, II) The bacterial seed endophyte communities differ within seeds of the same cultivar depending on the origin of the mother plants, III) *Rhizoctonia*-tolerant and –susceptible cultivars enrich different bacterial taxa in their rhizosphere, IV) *Rhizoctonia*-tolerant and –susceptible cultivars enrich similar taxa from different substrates, and V) *Rhizoctonia* tolerance can be mediated in susceptible cultivars by seed treatment with bacterial biocontrol agents. Our aim was to investigate bacterial communities in sugar beet and correlate them to the variables cultivar, seed origin, *Rhizoctonia* susceptibility and growth substrate.

## Material and Methods

### Experimental Setup

We analysed the naturally composed seed microbiome (“seed”), the roots of soilless germinated seedlings (“*in vitro”*) and roots of seedlings grown in different substrates (“*in vivo”*). Five sugar beet cultivars were chosen based on their phenotypic characteristics regarding the susceptibility towards the fungal phytopathogen *Rhizoctonia solani* (J.G.Kühn), the causative agent of late root rot in sugar beet; two susceptible cultivars [BELLADONNA KWS (BEL), BERETTA KWS (BER)], one moderately tolerant [ISABELLA KWS (ISA)] and two cultivars with partial resistance and tolerance [LAETITIA KWS (LAE]) MATTEA KWS (MAT); *Rhizoctonia*-tolerant cultivars]. Seeds of every cultivar were provided by KWS SAAT SE & Co. KGaA (Einbeck, Germany) from two different seed production sites, one in situated in France (Departement Lot-et-Garonne), one in Italy (Region Emilia Romagna). All samples were repeated 4 times.

For seed samples, 20 seeds per replicate were washed three times with sterile distilled water and activated for 4 h in 2 ml water. Sugar beet seeds were not surface-sterilized to simulate field-like conditions. For soilless cultivated sugar beet seedlings (“*in vitro”*), eight activated seeds were placed in germination pouches (Mega International, Newport, MN, USA) with two pouches per replicate. To avoid decoupling the rhizosphere microbiome from plant selection and to avoid training effects from agricultural field management including chemical compounds like fertilizer, pesticides, insecticides, and disseminated gene pools, we used potting soil instead of field soil for rhizosphere sampling. Nine activated seeds were placed in 7 cm × 7 cm × 13 cm pots with a soil:sand:vermiculite 3:1:1 (v/v) mixture, further denoted as “potting soil” (for details see **Supplementary Table 1**). To investigate the effects of natural-based biofertilizer on rhizosphere diversity, the setup was repeated for BEL, BER, LAE, and MAT using a natural product based on earthworm casts (**Supplementary Table 1**), further denoted as “vermicompost”. Vermicompost was used either as sole substrate or as amendment to potting soil. For the latter approach, ca. 15 g vermicompost was folded in the upper few centimetres of the potting soil before seeds were planted. Growing conditions were 23°C, 60% humidity and 16:8 h light/dark conditions for 2 weeks in all approaches.

### DNA Extraction

Activated seeds and *“in vitro”* roots weighed and grounded with 2 to 5 ml sterile 0.85% NaCl with mortar and pestle under sterile conditions. Suspensions were centrifuged at 16,500 × g for 20 min at 4°C and resulting pellets were stored at -70°C for further DNA extractions. DNA was extracted by mechanical disruption and homogenization of the pellet using and a FastPrep Instrument for 30 s at 5.0 ms^-1^ and FastDNA Spin Kit for Soil (MP Biomedicals, Illkirch, France). DNA was purified using GeneClean Turbo Kit (MP Biomedicals, Illkirch, France) to remove humic acids. Extracted DNA was treated with RNase (0.02 ng μl^-1^) for 5 min at 65°C to obtain the template for PCR amplification of 16S rRNA genes from total community DNA.

### Isolates From Sugar Beet and Vermicompost

Bacterial strains were isolated from vermicompost and sugar beet rhizospheres to compare culture-dependent and culture-independent results for the bacterial communities. For vermicompost and potting soil, 1 g substrate was diluted in 9 ml sterile 0.85% NaCl, vortexed, centrifuged at 16,500 g and 4°C for 20 min. For rhizosphere, supernatants of the suspensions used for amplicon sequencing were used. Bacteria were grown on R2A (Roth, Karlsruhe, Germany) at 30°C for 48 h. Clean CFUs were randomly chosen, isolated and grown on NA (Sifin GmbH, Berlin, Germany). Bacterial DNA was extracted using a “quick and dirty” protocol using a microwave for cell disruption; bacterial material was transferred to a 1.5 ml Eppendorf and rayed by a microwave for 3 min with closed lid, 30 µl TE buffer was added, vortexed and centrifuged at 16,500 x g for 2 min. Supernatant was used as template for a PCR using the bacterial universal primer pair 27F/1492R according to Lane et al. (1991). Amplifications were confirmed by gel electrophoresis in 1x TAE (0.8% Agarose). PCR-products were purified using Wizard SV Gel and PCR Clean-Up System (Promega, Madison, WI, USA). Sequencing was conducted by LGC Genomics (Berlin, Germany). Resulting sequences were quality-checked using Seq Scanner 2 (Applied Biosystems) and identified with BLAST (https://blast.ncbi.nlm.nih.gov/) using refseq_rna and/or nr/nt database.

### Amplicon Sequencing

The hypervariable V4 region of the 16S rRNA gene was amplified according to the protocol of Caporaso et al. (2011) using the primer pair 515f and 806r including Illumina cell flow adapters and sample-specific barcodes. Peptide nucleic acid (PNA) PCR clamps (PNA Bio, Newbury Park, USA) were used to reduce plastid and mitochondrial 16S contamination (Lundberg et al., 2013). The PCR mix (30 µl) contained 1 × Taq&Go (MP Biomedicals, Illkirch, France), 0.2 mM of each primer, 1.5 µM activated (55°C for 5min) PNA mix (1:1 mPNA:pPNA) and 1 µl template DNA. PCR conditions were 96°C for 5 min; 30 cycles of 96°C for 1 min, 78°C for 5 s, 54°C for 1 min, 74°C for 1 min; 74°C for 10 min). Amplifications of the resulting 168 samples were confirmed by gel electrophoresis in 1x TAE (0.8% Agarose). PCR-products were purified using Wizard SV Gel and PCR Clean-Up System (Promega, Madison, WI, USA). DNA concentrations were measured using Nanodrop 2000 (Thermo Scientific, Wilmington, USA) and equimolar aliquots of all samples were pooled for amplicon sequencing using an Illumina MiSeq v2 (250 bp paired end) platform conducted by LGC Genomics (Berlin, Germany).

### Bioinformatics

Pre-processing and analysis of the sequencing data was performed in QIIME2 v. 2019.10 (Bolyen et al., 2019) and QIIME v. 1.9.1 (Caporaso et al., 2010). Paired sample reads were demultiplexed and primers were trimmed from sequences using cutadapt (Martin, 2011). Since a considerable amount of forward and reverse sequences were flipped, reverse reads were also trimmed to forward primers and *vice versa*, and the resulting data from both trimming steps was combined. The sequences were denoised using DADA2 (Callahan et al., 2016), aligned with MAFFT (Katoh, 2002) and their phylogeny was constructed with fasttree2 (Price et al., 2010). Taxonomy was assigned with VSEARCH algorithm (Rognes et al., 2016) and Silva 132 99% consensus database (Quast et al., 2013). Mitochondrial, plastid DNA, and taxonomically unassignable sequences were removed from table and representative sequences. Thus, the datasets contained 32,140 amplicon sequences variants (ASV) and read numbers ranging from 254 to 545,913 reads per sample. Seeds and *in vitro* roots were separately analyzed from rhizosphere and soil. Alpha (Shannon, observed OTUs and evenness) and beta diversity indices (Bray-Curtis dissimilarity, weighted UniFrac) were calculated in QIIME2 and visualized using the q2_emperor plugin (Vázquez-Baeza et al., 2013). Differences in alpha and beta diversity indices were calculated using Kruskal-Wallis and pairwise PERMANOVA implemented in QIIME2. To evaluate taxa that differ significantly regarding cultivar and *Rhizoctonia* tolerance, samples of seed, *in vitro*, and *in vivo* roots were rarefied according to their minimum read number [1,000, 2,400 (losing 2 samples) and 15,000, respectively]. Samples for ISA were removed in *Rhizoctonia* tolerance testing to perform pairwise comparisons. The remaining samples were collapsed to genus level and compared using the QIIME1 plugin group_significance.py with Kruskal-Wallis test (cultivar, substrate) and nonparametric t-test (*Rhizoctonia* tolerance, seed origin). Differences between Bray-Curtis distances and weighted UniFrac distances of seeds and roots were tested using the adonis (Anderson, 2001) command of the R script vegan 2.5 (Oksanen et al., 2018) implemented in QIIME2.

To evaluate number proportion of surviving seed endophytes, seed and *in vitro* root samples were compared on ASV level. Intersection of communities of seed, soil, and rhizosphere were compared on genus-level with mean values of relative sequences.

### Field Design

Field trials were conducted in the course of efficacy tests in different years for the sugar beet cultivars BERETTA KWS (*Rhizoctonia*-susceptible, growing season 2009 and 2010), ISABELLA KWS (moderately tolerant, growing season 2016), and MATTEA KWS (*Rhizoctonia*-tolerant, growing season 2013). However, all field trials were performed under usual production conditions by local farmers. All sugar beet (*Beta vulgaris* L.) seed materials were generated and evaluated by KWS SAAT SE & Co. KGaA (Einbeck, Germany). Bacterial treatment was performed with a consortium of *Pseudomonas poae* RE*1-1-14 obtained from sugar beet (Zachow et al., 2010), *Pseudomonas brassicacearum* L13-6-12 (Grosch et al., 2005), and *Serratia plymuthica* 3Re4-18 (Berg et al., 2005) isolated from potato, which all show antagonistic activity towards *Rhizoctonia solani*. Formulations were prepared by Biotenzz GmbH (Graz, Austria) for integration in the commercial seed coating. Sugar beet seeds of *R. solani*-susceptible cultivars were routinely coated with the fungicides Thiram^®^ (Cheminova Deutschland GmbH, Germany), Hymexazol^®^ (Mitsui Chemicals, Tokyo, Japan) and the insecticides Imidacloprid (Gaucho^®^, Bayer CropScience, Leverkusen, Germany), Chlothianidin (Poncho^®^, Bayer CropScience, Leverkusen, Germany), Thiamethoxam (Syngenta Crop Protection, Basel, Switzerland), and Tefluthrin (Syngenta Crop Protection, Basel, Switzerland) respectively. The two test fields (“Kasten”: 48°42’48.6”N 13°04’34.5”E and “Tabertshausen”: 48°44’24.5”N 12°52’55.2”E) for the trials were located in the main growing area in Lower Bavaria, Germany. *R. solani*-infested barley kernels were used for artificial inoculation of the soil (60 kg/ha). Control plants were commercially prepared without microbial inoculants. Field trials were conducted in a randomized block design with four replicates per variant, each with six rows containing 30 plants per row. The *Rhizoctonia* disease index (RI) described by Büttner and coworkers (2004) with 1 to 9 (1—no disease, 9—plant dead, root completely rotted) and the number of beets were evaluated at harvest. Beets of the categories RI = 1 or 2 were defined as healthy beets. Statistical analysis was performed using SPSS version 25.0 (IBM Corporation, NY, USA). Correlation of number of beets at harvest, RI and percentage of healthy beets with bacterial treatment were tested pairwise (treated:untreated) for each cultivar and field separately using Mann-Whitney-U Test.

The 16S rRNA sequence of *Pseudomonas poae* RE*1-1-14 (NCBI Reference Sequence: NC_020209.1), *Pseudomonas brassicacearum* L13-6-12 (CP014693.1), and *Serratia plymuthica* 3Re4-18 (CP012097.1) was extracted from their reference genome and cross-checked with the amplicon dataset to evaluate presence or absence in our amplicon data samples.

## Results

### Seeds Harbor Cultivar-Specific Bacterial Communities Strongly Influenced by Propagation Sites

**Figure 1:**
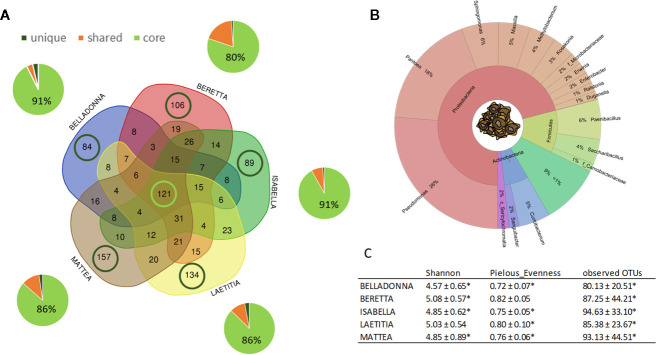
Overview on microbial community of sugar beet seeds. **(A)** number (VENN diagram) and corresponding abundance (pie charts) of amplicon sequence variants (ASVs) found in sugar beet seeds. Light green: seed core, found in all cultivars; dark green: cultivar-specific ASVs; orange: partially shared ASVs. **(B)** OTUs accounting for >1% relative abundance in the sugar beet seed microbial community on phylum and genus level. C: Alpha diversity indices of seed community; * = significantly differing according to Kruskal-Wallis test depending on seed origin (see also **Figure 2**).

Sugar beet seeds of five different cultivars originating from two different seed production sites contained a total of 1001 amplicon sequences variants (ASVs). For seed samples, read numbers ranged from 1,062 to 152,738 reads per sample (for a summary, see **Supplementary Table 2**). Seeds of all cultivars share a core of 121 ASVs, which accounts for 80%–91% rˤ (relative abundance) in the communities, while partially shared ASVs account for 4%–19% (**Figure 1A**); ASVs that are unique to a cultivar account for less than 4% rˤ (See also **Supplementary Table 3**). The seed endophyte community is dominated by *Pseudomonas*, *Pantoea*, and *Paenibacillus* (**Figure 1B**). Significant differences in alpha diversity indices (Shannon, Pielou’s evenness, observed OTUs) were not observed in seeds, except BER shows higher values for evenness than BEL and ISA (both p = 0.027). On the contrary, PERMANOVA results for differences in beta diversity indices (Bray-Curtis dissimilarity, weighted UniFrac distances) revealed to be largely significant (except: weighted UniFrac of BER:LAE; ISA:LAE). Main drivers of cultivar-dependent differences (rˤ > 1%) in seeds were *Pseudomonas* spp., *Paenibacillus* spp. and *Massilia* spp. (p<0.05; Bonferroni-corrected).

**Figure 2:**
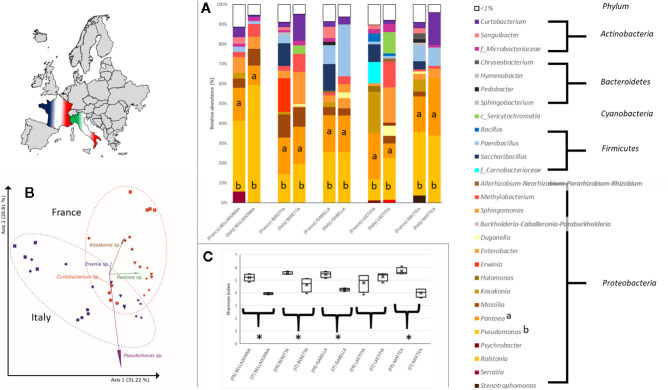
Differences in the bacterial community in sugar beet cultivars’ seeds due to origin. **(A)** Seed microbial community on genus level. All seeds are dominated to a different extent by *Proteobacteria*, especially *Pseudomonas* (a) and *Pantoea* (b). **(B)** PCoA-biplot of Bray-Curtis distance on genus level. Seed communities clearly cluster according to seed origin; spheres: cv. BELLADONNA; diamonds: cv. BERETTA; triangles: cv. ISABELLA; squares: cv. LAETITIA; stars: cv. MATTEA. **(C)** comparing Shannon diversity in seeds within the same cultivar due to seed origin; all seeds originating from France show a higher alpha diversity except cv. LAETITIA; * = significantly differing according to Kruskal-Wallis test depending on seed origin.

Seed origin was an important variable for alpha diversity (**Figures 1C**, **2A–C**), pairwise comparison within the same cultivars revealed significant differences (pairwise Kruskal-Wallis) for observed OTUs (all) and Shannon index (except LAE). Additionally, all cultivars differed significantly (p < 0.05) due to seed origin in Bray-Curtis and weighted UniFrac distances. Adonis test of Bray-Curtis and weighted UniFrac distances revealed the factors “Cultivar” and “Seed origin” explain more than 50% of the variance (**Supplementary Table 4**), with “Cultivar” being the more important one (R^2^ = 0.33 and R^2^ = 0.32, respectively; Pr(>F) = 0.001). Key genera that significantly differ due to seed origin are not the same for the different cultivars **(** **Supplementary Table 5**). When merging all seed data, *Sphingomonas*, *Methylobacterium* and unidentified *Sericytochromatia* show higher rˤ-values in Italian seeds, while *Saccharibacillus*, *Kosakonia*, and *Erwinia* have higher proportion in French seeds; this is also indicated by PCoA-biplot of Bray-Curtis distances (**Figure 2B**).

### Dominating Seed Endophytes Survive Host Germination

**Figure 3:**
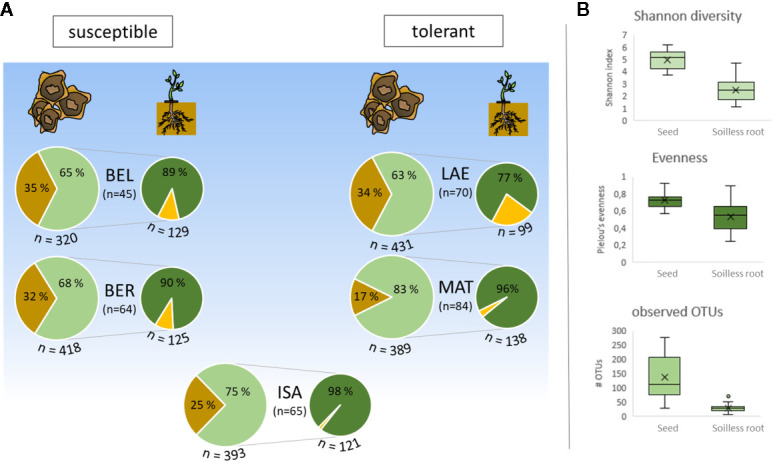
Comparing seed and *in vitro* root microbial community on relative abundance of sequence variants (ASV). **(A)** fraction of surviving bacteria originating from seeds (left pie chart) in soilless seedlings (right pie chart). Percent values refer to mean relative abundance, while n stands for the number of involved ASVs (below pie charts: total number in microhabitat; between pie charts: number of shared ASVs); brown: unique in seeds; green: shared ASVs; yellow: unique *in vitro* root ASVs. **(B)** Differences in alpha diversity indices between seed and *in vitro* root communities.

To assess the proportion of alive bacterial communities within seeds, 20 seeds each cultivar were cultivated under *in vitro* conditions in soilless germination pouches. The *in vitro* rhizosphere contained 397 ASVs, represented by 2,533,778 reads, with read numbers ranging from 254 to 137,909 reads per sample. Depending on cultivar, ASVs accounting for 63%–83% rˤ found in the seeds could be confirmed to be present in soilless roots (*“in vitro*”, **Figure 3A**).

Dominant phyla in soilless roots are *Proteobacteria* (range 75%–91% rˤ, depending on cultivar), *Actinobacteria* (1%–18% rˤ), and *Firmicutes* (2%–22% rˤ). *Enterobacteriaceae*, mainly represented by *Kosakonia* (19% rˤ) and *Pantoea* (18% rˤ) account for 40% rˤ in soilless roots, although *Methylobacterium* (28% rˤ) is the most frequent genus.

The number and proportion of ASVs of successfully colonizing bacteria differ between cultivars (n = 45–70, ≙ 14%–22% of corresponding seed ASVs), but represent dominating taxa of seed endophytes. Tendentially, a higher proportion of seed endophytes survive in soilless roots of *Rhizoctonia*-tolerant cultivars (14%–15% vs. 16%–21% of corresponding seed ASVs; **Figure 3A**). In all cultivars investigated, *in vitro* roots display a lower alpha diversity and a lower evenness (**Figure 3B**) than the corresponding seeds. ASVs representing up to 14% rˤ (for LAE) in soilless roots were found to be unique and thus of unknown origin. In total, 230 ASVs unique to *in vitro* roots were found across all cultivars with 12 ASVs accounting for rˤ >1% per sample. These ASVs belong to the genera *Curtobacterium*, *Bacillus*, *Pullulanibacillus* and *Methylobacterium*, taxa that are frequently present in both seeds and *in vitro* roots. Cultivar-dependent differences for alpha diversity (Shannon) were not found, and beta diversity indices (Bray-Curtis, weighted UniFrac) were only significantly different between BEL and MAT.

### The Ingredients of the Rhizosphere: Soil and Seed Bacteria

**Figure 4:**
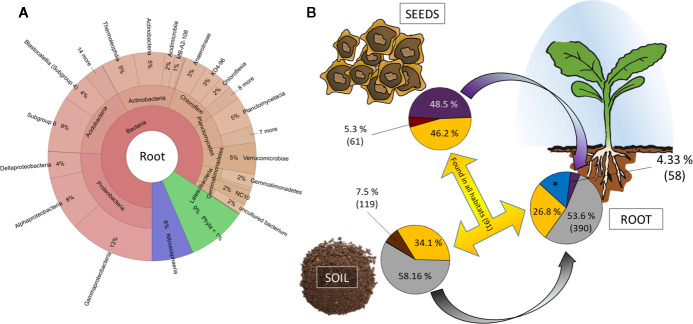
Tracking the origin of bacterial genera found in the rhizosphere. **(A)** Krona chart of root communities on phylum level. Abundances are mean values across al cultivars. **(B)** Relative abundance and number of genera unique (soil: brown; red: seed; blue: root) and shared (yellow) between habitats. *: 417 genera accounting for 15.25% relative abundance exclusively found in rhizosphere and with thus unknown origin.

The *in vivo* root community clearly differs from both soil and seed communities and is dominated by *Proteobacteria* (26% rˤ), *Acidobacteria* (16% rˤ), *Actinobacteria* (13% rˤ), *Chloroflexi* (10% rˤ), and *Planctomycetes* (7% rˤ; **Figure 4A**). When the dataset was collapsed to the highest taxonomic annotation, the dominant taxa were *Acidobacteria* Subgroup 6 (7.0% rˤ), the archaeal family *Nitrososphaeraceae* (3.9% rˤ), and the genus *Pyrinomonadaceae* RB41 (3.9% rˤ). Differences in root beta diversity due to seed origin were not significant. The bacterial core community of *in vivo* roots comprised of 3,228 ASVs, representing 82%–88% rˤ depending on cultivar. Cultivar-specific ASVs account for 2.2%–3.1% of the abundance (**Supplementary Table 3**).

The bigger part of the root community is originating from soil. Genera accounting for 53.6% are exclusively assembled from soil and another 26.8% of the root community comprises taxa that are found in both seeds and soil (**Figure 4B**). Only a small proportion of the seed and soil microbiome (5.3% and 7.5%, respectively) is unique to their corresponding habitats, while we find 417 genera accounting for 15.25% of the rhizosphere microbiome to be unique. The key taxa found in seeds appear in lower relative abundances in rhizosphere. From the genera that exclusively originate from seeds, four are found in >50-fold rˤ-values in root samples; 41 of soil-derived taxa are >50-fold increased (**Supplementary Table 6**).

Interestingly, *Archaea* were found in high abundances (up to 14.1% rˤ) in the root and vermicompost-associated communities, with *Nitrososphaeraceae* (ad *Thaumarchaeota*) as dominant taxa (in average representing 91% of all archaeal taxa).

### Indicator Bacteria for *Rhizoctonia*-Tolerant Cultivars

ASVs, that are exclusively found in seeds of *Rhizoctonia*-tolerant cultivars (n = 20) account for 0.83% rˤ (LAE) and 0.88% rˤ (MAT). When combining seeds, potting soil and roots samples, adonis test revealed the factor “habitat” to be the most important variable explaining the variance for Bray-Curtis and weighted UniFrac distances (R^2 ^= 0.421 and R^2 ^= 0.725, respectively). The factors “seed origin” and “*Rhizoctonia*-tolerance” explain approximately the same amount of variance in both Bray-Curtis (R^2^ = 0.029 and R^2^ = 0.027) and weighted UniFrac distances [R^2^ = 0.014 and R^2^ = 0.015, respectively (**Supplementary Table 7**]. When exclusively testing root samples (seed origin = Italy) grown in the three different substrates, the factor “*Rhizoctonia*-tolerance” explains a higher percentage of variance than the factor “Cultivar” for both beta diversity indices (**Supplementary Table 8**).

**Figure 5:**
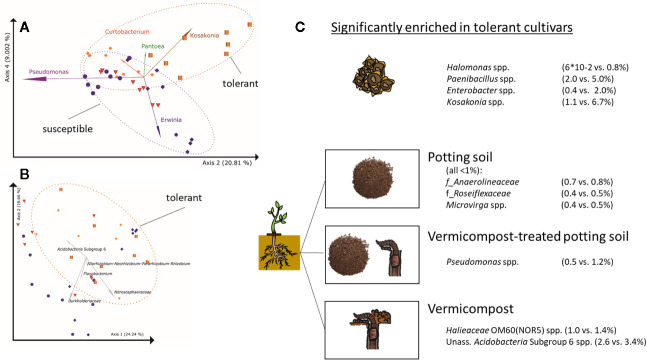
Differences in bacterial root communities between *Rhizoctonia solani*-susceptible (violet), effective (red) and –tolerant (orange) cultivars **(A)** PCoA-biplot of Bray-Curtis dissimilarities in seeds on genus level; spheres: cv. BELLADONNA; diamonds: cv. BERETTA; triangles: cv. ISABELLA; squares: cv. LAETITIA; stars: cv. MATTEA. Note, that PCoA axes 2 and 4 separate the communities based on *Rhizoctonia* tolerance, while axes 1 and 2 separate them due to their origin(**Figure 2B**). For the visualization of the other combinations of PCoA axes, see **Supplementary Figure 1**. **(B)** PCoA-biplot of Bray-Curtis dissimilarities in roots on genus level; **(C)** Taxa significantly enriched in *Rhizoctonia* tolerance cultivars (genus level) in seeds and roots grown in potting soil, vermicompost-treated potting soil and vermicompost.

Taxa significantly and positively correlated with *Rhizoctonia*-tolerance in seeds are *Halomonas* spp., *Paenibacillus* spp, *Enterobacter* spp., and *Kosakonia* spp. (**Figure 5A**), while *Massilia* spp. is higher in seeds of susceptible cultivars. In soilless roots, *Firmicutes*, mainly represented by *Paenibacillus* (except for LAE: *Pullulanibacillus* and *Bacillus*), are higher abundant in *Rhizoctonia*-tolerant cultivars than in susceptible ones, and *vice versa* for *Actinobacteria* (**Supplementary Table 9**), mainly represented by *Curtobacterium*. For the root samples, clustering of samples in PCoA plots due to *Rhizoctonia*-tolerance is less pronounced than in seeds (**Figure 5B**). A general trend across seeds, *in vitro*, and *in vivo* root samples is the higher abundance of *Firmicutes* in *Rhizoctonia*-tolerant cultivars.

Root communities of *Rhizoctonia*-tolerant cultivars show a trend (non-significant) towards higher rˤ-values of *Gaiellales* (ad *Actinobacteria*) and *Rhizobiales* (ad *Proteobacteria*). rˤ-values of *Bacteroidetes* and *Verrucomicrobia* are significantly (p = 0.02) higher in roots of susceptible.

Genera significantly higher in *Rhizoctonia*-tolerant cultivars differ according to used substrates (**Figure 5C**). However, the genus *Kosakonia*, which is one of the taxa correlated with *Rhizoctonia* tolerance in seeds, tends to be higher abundant in *Rhizoctonia*-tolerant root samples across all substrates.

### The Impact of Substrates on Root Microbiota

**Figure 6:**
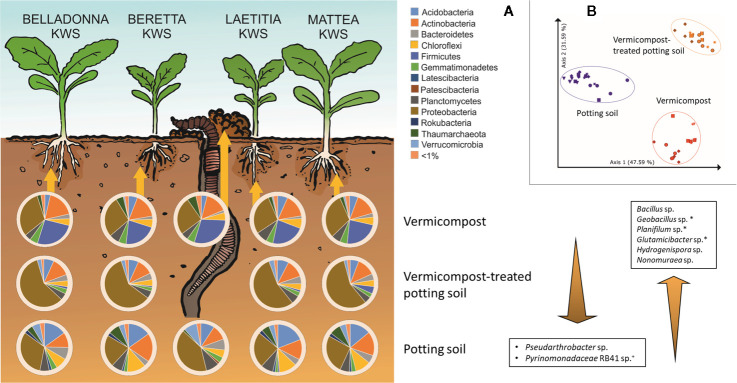
Influence of different substrates on bacterial root communities in sugar beet. **(A)** Composition of bacterial root communities in different substrates on phylum level. Bacterial genera displayed that are significantly different (Bonferroni-corrected p < 0.05) between substrates, have >1% mean relative abundance and have intermediate abundance in VC-PS samples. ^+^: unique for potting soil; *: unique for vermicompost; PS: potting soil; VC-PS: vermicompost-treated potting soil; VC: vermicompost. **(B)** PCoA-plot of Bray-Curtis dissimilarities of root samples grown in different substrates, only samples with seed origin from Italy; spheres: cv. BELLADONNA; diamonds: cv. BERETTA; squares: cv. LAETITIA; stars: cv. MATTEA.

Root samples of BEL, BER, LAE, and MAT grown in the three different substrates (potting soil, vermicompost and mixtures) were merged in a data subset. Differences in the overall composition of root microbiomes are already apparent in high taxonomic ranks. Vermicompost addition to potting soil highly increases rˤ of *Proteobacteria* across all cultivars. *Firmicutes* and *Bacillus* spp. in particular are found in high abundance in pure vermicompost (**Figure 6A**). Results for pairwise Kruskal-Wallis test between “substrate” groups were filtered for genera that a) have rˤ >1% in at least one of the substrate groups and b) show high rˤ-values in one of the pure substrates and intermediate in mix of vermicompost-treated potting soil. Six genera matching these prerequisites were thus shown to be gradually enriched with increased use of vermicompost (**Figure 6A**).

The factor “substrate” explains around 68% of the variance in both Bray-Curtis and weighted UniFrac distances (**Supplementary Table 8**). In PCoA visualization plots, samples clearly cluster due to substrate (**Figure 6B**).

### Cultivation-Dependent vs. -Independent Microbiome Assessment

Bacteria were isolated to find bioactive strains and compare cultivation-dependent to -independent approaches. We successfully isolated and identified 339 bacterial strains belonging to at least 137 different species from pure vermicompost (n =192; 73 species), roots grown in vermicompost-treated potting soil (n = 95; 41 species) and roots grown in pure vermicompost (n = 112; 56 species, **Supplementary Table 10**). The most frequently isolated species associated with vermicompost was *Agromyces flavus*. Additionally, we found 62 strains of at least 20 different *Bacillus* species, with *B. firmus* (n = 15) being the most frequently isolated species. Furthermore, the diversity of *Microbacterium* spp. (n = 19; six species) and *Streptomyces* spp. (n = 12; 9 species) is worth mentioning.

**Figure 7:**
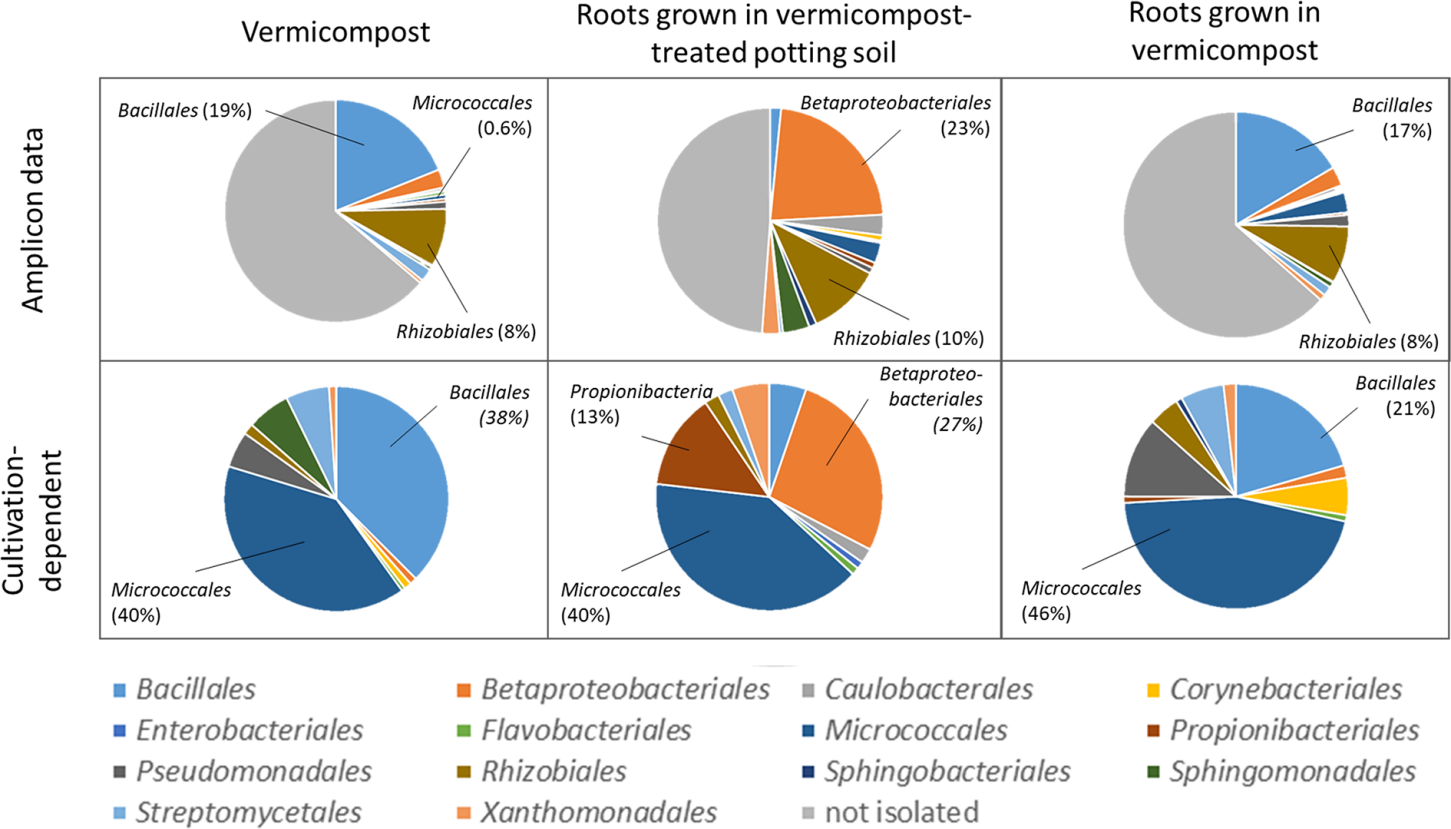
Comparison of microbiome assessment using cultivation-dependent (isolation on R2A plates) and –independent (16SrRNA amplicon sequencing) approaches on order level.

Results of cultivation-dependent assessment clearly differ from amplicon data. On order level, a third up to half of all orders present in the amplicon data could be cultivated. When comparing rˤ-values between cultivated strains and amplicon data, the most apparent difference is the high proportion of *Micrococcales* in the isolate dataset across all three groups. In contrast, sequences of *Micrococcales* account for rˤ <3% to the amplicon dataset. (**Figure 7**). The dominance of *Bacillales* in vermicompost and *Betaproteobacteriales* in roots grown in soil-vermicompost mix is confirmed by both amplicon and cultivation data. *Rhizobiales* reached higher rˤ-values in the amplicon dataset across all groups.

### Inducing *Rhizoctonia* Tolerance Using a Consortium Containing *Ps. poae* Re*1-1-14

Sugar beet seeds with standard fungicide and were compared to a combined treatment with both bacterial consortium and fungicide treatment. The percental change in RI, total number of beets and number of healthy beets due to bacterial treatment were compared. Statistical pairwise comparisons did not result in significant differences (p > 0.05), most likely due to the patchy infestations of *Rhizoctonia* observed in the field and subsequently high standard deviation in the dataset. Nevertheless, trends towards different responses due to cultivar were observed: the application of the bacterial consortia showed strongest effects in the *Rhizoctonia*-susceptible cultivar, especially at the field with higher soil humidity and thus higher pathogen pressure (“Kasten”). Under these conditions a mean RI decrease of 26% as well as an increase in the number of healthy sugar beets of 65.4% relative to standard treatment could be achieved (**Supplementary Figure 2**) in susceptible cultivars. For tolerant or moderately tolerant cultivars, positive effects regarding RI and number of healthy beets were less pronounced or not observed. In average, the total number of sugar beets at the time of harvest increased by 5% using bacterial treatment.

## Discussion

We analyzed microbial communities in seed, roots and corresponding soil to investigate sugar beet microbiota assembly and composition. We found novel aspects, which can be translated to manage the plant microbiome. The bacterial root community of sugar beet derives from both seed and soil communities. Seeds of all sugar beet cultivars were highly colonized by bacteria and carried a selective core of the sugar beet microbiome, which contributed significantly to the rhizosphere assembly. We found relatively high alpha diversity values for the sugar beet seeds, with results comparable to pumpkin seeds (Adam et al., 2018) but significantly lower than in sugar beets at harvest (Kusstatscher et al., 2019). The high proportion of *Proteobacteria, Actinobacteria, Firmicutes* and *Bacteroidetes* found in sugar beet seeds is quite typical for seed microbiota (reviewed by Nelson, 2018). However, especially *Enterobacteriaceae* were identified as important component in seeds. For instance, *Enterobacteriaceae* and in particular *Klebsiella*, dominate in pumpkin seeds (Adam et al., 2018). *Pantoea*, one of the key taxa in sugar beet seeds, was also found to be dominant in *Brassica* seeds (Barret et al., 2015a) except for oilseed rape (Rybakova et al., 2017). *Pantoea* comprises versatile lifestyles, including plant pathogens, plant growth promoters as well as strains commercially produced for biocontrol of phytopathogens. Thus, *Pantoea* is a model group for niche-specific adaptations (Walterson and Stavrinides, 2015). Furthermore, abundance of some *Enterobacteriaceae* (*Kosakonia* and *Enterobacter*) together with *Paenibacillus* are correlated with *Rhizoctonia* tolerance (**Figures 5A, C**) in sugar beet seeds. *Paenibacillus* is known to contain species with plant growth-promoting and pathogen-antagonistic properties (reviewed by Rybakova et al., 2016). Similarly, some *Enterobacter* species show antagonistic properties against *Rhizoctonia* (e.g. Abdeljalil et al., 2016). *Kosakonia radicinitrans* is known for both plant growth promotion and indirectly reducing pest pressure in *Arabidopsis thaliana* (Brock et al., 2018). Nevertheless, bacterial seed communities rather cluster due to origin (**Figure 2B**, displaying PCoA axes 1 and 2) than to *Rhizoctonia* tolerance (**Figure 5B**, displaying PCoA axes 2 and 4) of the cultivars.

One interesting fact is the higher abundance of different groups of *Firmicutes* (*Pullulanibacillus* in LAE, *Paenibacillus* in MAT, see **Supplementary Table 9**) in seeds of *Rhizoctonia*-tolerant cultivars. This indicates that the function provided by several different groups of bacteria is more important for the plant-microbe interaction than their exact taxonomic position; taxa significantly solely enriched in *Rhizoctonia*-tolerant cultivars account for less than 1% rˤ. Although our study suggests which taxa could be correlated with *Rhizoctonia* tolerance, we are aware that five genotypes are not sufficient to suggest a general trend for microbial shifts due to breeding. This also applies for our results for *in vitro* roots and *in vivo* roots. However, other authors found different taxa associated with response upon fungal invasion with *R. solani* than we found in higher abundances in tolerant cultivars (Chapelle et al., 2016; Carrión et al., 2019).

This study is the first one to track seed-associated bacteria during germination at sequence level and correlate microbial communities to differences in *Rhizoctonia* tolerance. The majority of seed endophytes is able to survive the process of germination, although speaking of species number they only represent a subset of seed-associated microbiota (**Figure 3A**). These species are thus available for the next sugar beet generation, confirming vertical transmission of seed endophytes in sugar beet. Apparently, the proportion of surviving endophytes may still be underestimated due to our experimental setup, including higher exposure of the roots to oxygen and light compared to conditions in soil. This may apply for seed endophytic *Archaea*, which are found in 50% of seed samples but only in 35% of *in vitro* root samples. However, the bacterial community is shifted in the course of germination, which was also observed in *Brassica* plants (Barret et al., 2015b), wheat (Huang et al., 2016) and maize (Johnston-Monje et al., 2016). When focusing on general aspects of the *Rhizoctonia*-tolerant cultivars’ microbial seed communities during germination (**Figure 3A**), the most striking facts are, that a) a higher proportion (absolute and relative) of seed-associated bacteria in cv. ISABELLA and cv. MATTEA seeds are still represented in *in vitro* root samples, and b) cv. LAETITIA shows the highest seed alpha diversity across all tested cultivars. Although we know that bacterial alpha diversity is usually correlated with pathogen tolerance (van Elsas et al., 2012), a high diversity may not be necessary if members of the microbial community provide all important functions to the plant-microbe-pathogen interaction. We hypothesize this to be the case in cv. ISABELLA and cv. MATTEA since the majority of seed endo- and epiphytes survive in the germinated seedlings.

The drop in microbial communities’ diversity during germination (**Figure 3B**) is usually interpreted as selective force exerted by the seedling, favoring fast-growing r-strategists like *Pseudomonas*, *Bacillus* or *Trichoderma* (Barret et al., 2015a). In our dataset, *Methylobacterium* dominated soilless roots and they are regarded to be typical K-strategists due to their ability to metabolize C1-compounds. Therefore, we suggest the indirect selective force of the germinating host plants rather to favor seed endophytes that are adapted to their hosts’ specific genotype (Berg and Raaijmakers, 2018) or the present cultivation conditions (Bergna et al., 2018). Given that this turnover of the seed core is proportionally more or less stable over generations, there are niches in the seed microbiome that can be colonized with bacteria enriched from soil, shaping the seed microbiome of the next generations’ seeds (Bergna et al., 2018).

The soil microbiome can influence the rhizosphere microbiome composition of the seedlings and thereof influences the host (Nelson, 2018). We confirm soil as the main source of diversity in sugar beet rhizosphere. Nevertheless, a considerable amount of rhizosphere-inhabiting bacteria exclusively originates from seeds, and 26.8% of all bacterial genera could be provided by either bacterial soil or seed endophyte communities. Interestingly, 417 genera accounting for 15.3% of the rhizosphere community could not be traced back to either seed or soil (**Figure 4B**). The majority of these genera have low rˤ-values (<0.5%). We regard these members of the root microbiome to be enriched in rhizosphere but under the detection threshold in soil and seed samples. This explains why alpha diversity in roots (Shannon H = 9.61 ± 0.56) is significantly (p = 0.02) higher than in soil samples (Shannon H = 8.94 ± 0.05). The differences in microbial communities due to seed origin, cultivar and *Rhizoctonia* tolerance are more pronounced in seeds than in roots. *Roseiflexaceae* and *Anaerolineaeceae*, which are significantly enriched in *Rhizoctonia*-tolerant cultivars, are worldwide distributed taxa (GBIF.org, 2020), found in water as well as soil habitats and mainly comprise yet monotypic genera. The genus *Microvirga* on the other hand is frequently found in root nodules of legumes in temperate climates (e.g. Msaddak et al., 2017). Seed origin was an important variable for bacterial seed communities. One should consider that crops are usually planted across large geographical areas and location-dependent bacterial communities were already revealed in maize (Johnston-Monje and Raizada, 2013) and common beans (Klaedtke et al., 2016). Although community differences due to the origin of seeds in both alpha and beta seed diversity indices are obvious and apply to several different bacterial taxa (**Figures 2A–C**), the cultivar—and thus the genotype as well as the phenotype (e.g. root architecture) of the host plant—is the more important factor explaining the seed communities’ variance.

Considering earthworms, their ecosystem services and their microbes in agricultural practices holds a big potential for the agriculture of the future (Singh et al., 2020). The microbial community of vermicompost, as a product obtained mainly from organic litter earthworm casts, clearly differs from potting soil, with especially higher proportions of *Bacillus* (5.22 vs. 0.02%). High abundance of *Bacillus* spp. relates to the processing of organic matter in the gut of earthworms. In the course of digestion, the microbial community is exposed to several changes in conditions, including pH neutralization, higher water content, complete anoxia, secretion of digestive enzymes and enrichment of organic compounds including fermentation products from other microbes (reviewed by Drake and Horn, 2007). These conditions on the one hand favor facultative anaerobic or aerobic bacteria that are able to form endospores (Drake and Horn, 2007), and on the other hand activate endospores of *Bacillus* (Fischer et al., 1997). The genus *Bacillus* contains several species that are known for direct as well as indirect phytopathogen antagonism and plant growth-promoting effects (e.g. Pérez-García et al., 2011). We observed higher abundance of *Firmicutes*—in particular the genus *Bacillus*—in roots of *Rhizoctonia*-tolerant cultivars grown in untreated and vermicompost-treated potting soil. When using pure vermicompost as substrate, abundance of Bacillus was comparably high (4.1%–6.2% rˤ) in all cultivars. We interpret this as specific enrichment of pathogen-antagonistic bacteria by *Rhizoctonia*-tolerant sugar beet cultivars from the substrate. Since the abundance of *Firmicutes* is generally higher in vermicompost than in potting soil, enrichment in the rhizosphere may not be necessary to achieve pathogen suppression. Some basic information on the microbial community when using vermicompost as substrate are concordant in both cultivation-dependent and –independent approaches. This includes the dominance of *Bacillales* in vermicompost-associated samples and the dominance of *Betaproteobacteriales* in vermicompost-treated potting soil samples (**Figure 7**).

The effect of bacterial control agents in field trials was strongest for the *Rhizoctonia*-susceptible cultivar BERETTA, indicating a strong influence of the microbial community in the early stage of rhizosphere establishment. Applying bacterial control agents to *Rhizoctonia*-tolerant cultivars did not result in further increased, but partially showed slightly decreased health parameters. This may indicate interference of the applied control agents with native root bacteria. The bacterial treatment consisted of three strains, of which *Pseudomonas poae* RE*1-1-14 was originally isolated from sugar beet. This strain shows an interesting pattern across our dataset (**Supplementary Table 11**), since it is more frequently and in higher abundances in *Rhizoctonia*-tolerant cultivars. The genus *Pseudomonas* itself is frequently found across all life stages of sugar beet and several strains with promising biocontrol potential were found in sugar beet endosphere before (Zachow et al., 2008; Zachow et al., 2010). RE*1-1-14 was initially selected in a screening for antagonistic strains against several different sugar beet pathogens (*Phoma betae*, *R. solani* AG2-2IIIB, *R. solani* AG4 and *Sclerotium rolfsii*; Zachow et al., 2010). Despite the specific ASV was not exclusively found in *Rhizoctonia*-tolerant cultivars, *Ps. poae* seems to be an integral part of *Rhizoctonia*-tolerant cultivars and provide important plant-protecting functions to the seedling. Interestingly, *Ps. poae* is more frequently found in sugar beets originated from France, where sugar beets are grown on much larger geographical areas (EUROSTAT, 2019) and historically for a longer time span compared to Italian acreages. These factors are known to favor the development of soil communities suppressive to certain pathogens (Mazzola, 2002; Eberlein et al., 2020). Continuous cropping of sugar beet is known to influence the field soil community, accelerating the abundance of different taxa in the microbial soil community over time (Huang et al., 2020). We found partially cultivar-specific differences between root communities. Thus, continuous cropping of self-reproduced genotypes may shape both bacterial soil and seed endophyte communities, potentially leading to a cultivar-dependent enrichment of taxa that are beneficial and/or usually uncommon in seeds.

## Conclusions

Overall, found differences in the microbiomes of *Rhizoctonia*-susceptible and -tolerant sugar beet cultivars across various life stages. In seeds, the genus *Kosakonia* may play a role in pathogen tolerance. Seed communities in sugar beet differ due to seed origin and cultivar; differences are more pronounced than in seedling roots and rhizospheres, although substrate heavily determine root community structure. Further investigation with a higher number of different *Rhizoctonia*-susceptible and tolerant cultivars are needed for confirming general trends in sugar beet-associated bacterial communities with *Rhizoctonia*-tolerance. This investigation can be explored in more detail by implementing crop breeding strategies that include the trait “plant microbiome”. Future strategies for sustainable agriculture might be able to include microbiome management.: 1) Selection of seed production sites could include soil microbiome analysis as seeds take up sets of microbes differently involved in stress response in the next generation. 2) The crop/plant microbiome can be adjusted *via* seed treatments that add further microorganisms to the soil 3). The entire soil microbiome can be managed by microbiome transplants.

## Data Availability Statement

The datasets presented in this study can be found in online repositories. The names of the repository/repositories and accession number(s) can be found below: https://www.ebi.ac.uk/ena, PRJNA299370 https://www.ebi.ac.uk/ena, PRJEB37140.

## Author Contributions

AW, GB, CZ, and HM drafted the manuscript. CZ, AW, HM, AG, NT, RT, and GB planned the experimental setup. AW, CZ, and HM performed molecular, microbial, and *ad planta* work. AW conducted bioinformatic analyses. AW, CZ, HM, GB, NT, and RT either planned and/or performed the field trials. All authors contributed to the article and approved the submitted version.

## Funding

This work was funded by the Austrian Centre of Industrial Biotechnology (acib GmbH), which has been supported by the Austrian BMWFJ, BMVIT, SFG, Standortagentur Tirol, and ZIT through the Austrian FFG-COMET-Funding Program.

## Conflict of Interest

HM is employed by BioTenzz GmbH. NTand RT are employed by KWS SAAT SE & Co. KGaA. AG is employed atVERMIGRAND Naturprodukte GmbH. GB is employed at Graz University of Technology. AW and CZ were employed at the Austrian Centre of Industrial Biotechnology (acib GmbH) during the creation of this manuscript.

The remaining authors declare that the research was conducted in the absence of any commercial or financial relationships that could be construed as a potential conflict of interest. 

## Supplementary Material

Supplementary Figure 1 PCoA-biplots of sugar beet seed endophyte communities on genus level displaying Bray Curtis dissimilarities using PCoA axes 1 and 3 **(A)**, 1 and 4 **(B)** and 2 and 3 **(C)**.

Supplementary Figure 2 Results of field trials for relative in-/decrease of Rhizoctonia disease index (RI, according to Büttner et al., 2004), number of sugar beets and number of healthy sugar beets for the cultivars BER, ISA and MAT using a consortium of three bacterial antagonists additionally to standard fungicide treatment. Results are relative to sugar beet seeds with standard fungicide treatment. Comparisons did not result in significant differences but trends towards stronger positive effects in the Rhizoctonia-susceptible cultivar.

## Abbreviations

ASV, amplicon sequence variants; BEL, sugar beet cultivar BELLADONNA KWS, *Rhizoctonia*-susceptible; BER, sugar beet cultivar BERETTA KWS, *Rhizoctonia*-susceptible; cv, Cultivar; FR, France; ISA, sugar beet cultivar ISABELLA KWS, *Rhizoctonia*-effective (intermediate); IT, Italy; LAE, sugar beet cultivar LAEITIA KWS, *Rhizoctonia*-tolerant; MAT, sugar beet cultivar MATTEA KWS, *Rhizoctonia*-tolerant; PCoA, Principal coordinate analysis; rˤ, mean relative abundance, romanized version of ancient Egyptian: Ra, deity of the sun.
