# Supplementary figures and images for: Understanding the Impact of Cultivar, Seed Origin, and Substrate on Bacterial Diversity of the Sugar Beet Rhizosphere and Suppression of Soil-Borne Pathogens
Source: Front Plant Sci. 2020 Sep 30;11:560869. doi: 10.3389/fpls.2020.560869 (PMC7554574; doi:10.3389/fpls.2020.560869)

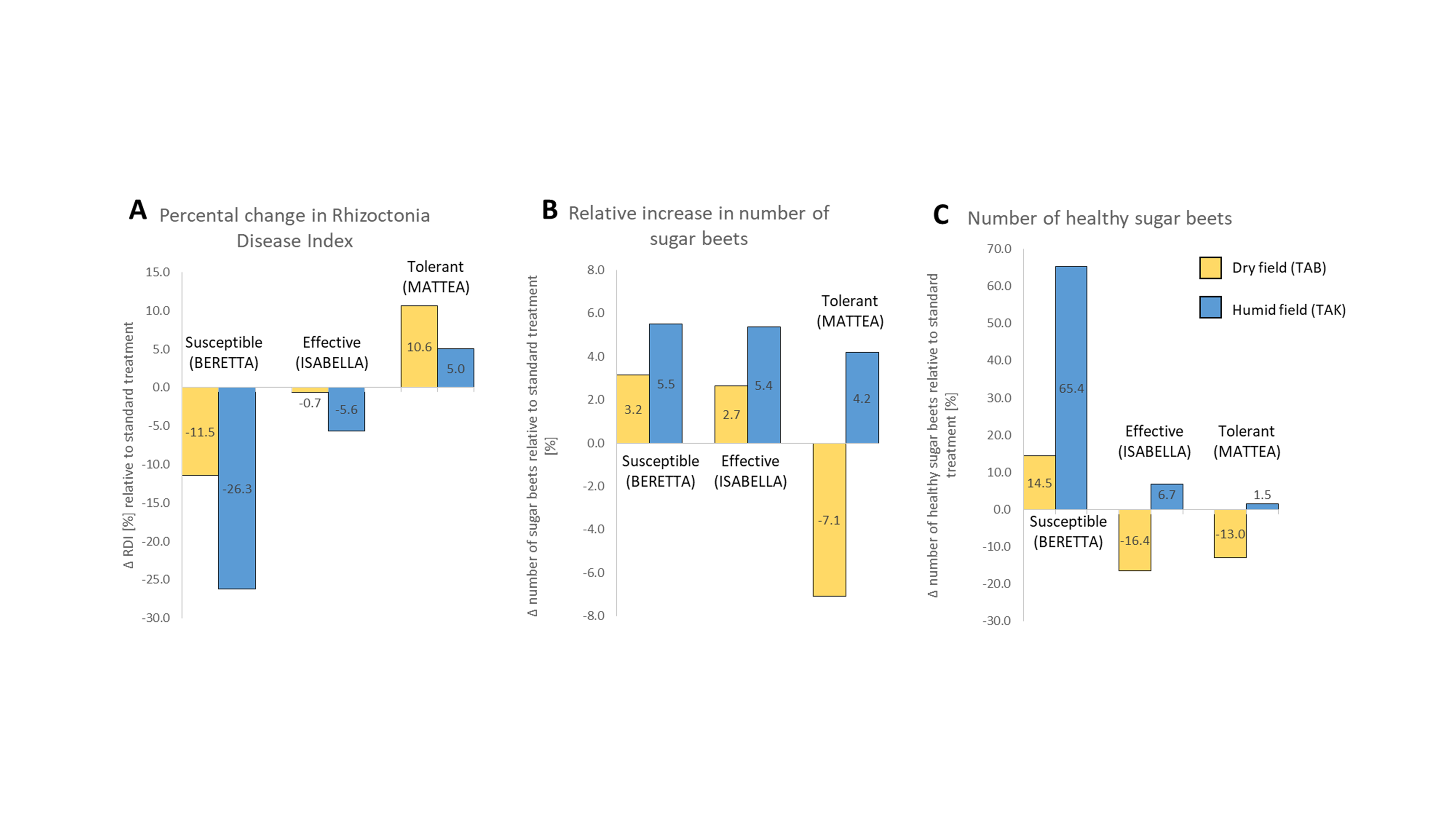

Supplement: Supplementary Figure 2 [file Image_2.tif]
